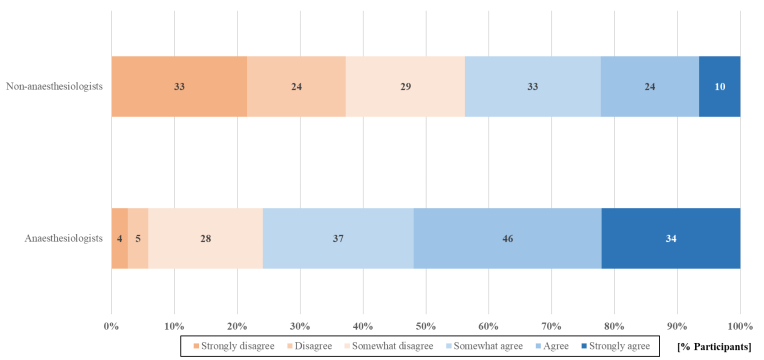# Local anaesthetics risks perception: A web-based survey

**DOI:** 10.1016/j.heliyon.2023.e23545

**Published:** 2023-12-13

**Authors:** Tal Sarah Beckmann, Caroline Flora Samer, Hannah Wozniak, Georges Louis Savoldelli, Mélanie Suppan

**Affiliations:** a Division of Anaesthesiology, Department of Anaesthesiology, Clinical Pharmacology, Intensive Care and Emergency Medicine, Geneva University Hospitals and Faculty of Medicine, Geneva, Switzerland; b Division of Clinical Pharmacology and Toxicology, Department of Anaesthesiology, Clinical Pharmacology, Intensive Care and Emergency Medicine, Geneva University Hospitals and Faculty of Medicine, Geneva, Switzerland; c Interdepartmental Division of Critical Care, University of Toronto, Toronto, Canada; d Division of Intensive Care, Department of Anaesthesiology, Clinical Pharmacology, Intensive Care and Emergency Medicine, Geneva University Hospitals and Faculty of Medicine, Geneva, Switzerland

**Keywords:** Local anaesthetics, Toxicity, Drug safety, Dose calculation, Web-based survey

## Abstract

**Background:**

The use of local anaesthetics (LAs) is usually associated with few adverse effects, but local anaesthetic systemic toxicity (LAST) can result in serious harm and even death. However, practitioner awareness regarding this risk has been little studied.

**Methods:**

This was a closed, web-based study carried out at two Swiss university hospitals using a fully automated questionnaire. The main objective was to evaluate LAST awareness and LA use among various medical practitioners. The secondary objective was to determine whether these physicians felt that a tool designed to compute maximum safe LA doses should be developed.

**Results:**

The overall participation rate was 40.2 % and was higher among anaesthesiologists (154/249, 61.8 % vs 159/530, 30.0 %; *P* < .001). Anaesthesiologists identified the risk of LAST and the systems involved more frequently than non-anaesthesiologists (85.1 % vs 43.4 %, *P* < .001). After adjusting for years of clinical experience, age, country of diploma, frequency of LA use, clinical position and being an anaesthesiologist, the only significant associations were this latter factor (*P* < .001) and clinical position (*P* = .016 for fellows and *P* = .046 for consultants, respectively). Most respondents supported the development of a tool designed to compute maximum safe LA doses (251/313, 80.2 %) and particularly of a mobile app (190/251, 75.7 %).

**Conclusions:**

LAST awareness is limited among practitioners who use LAs on a regular basis. Educational interventions should be created, and tools designed to help calculate maximum safe LA doses developed. The actual frequency of unsafe LA doses administration would also deserve further study.

## Highlights

• There is little scientific data regarding local anaesthetic systemic toxicity awareness among medical practitioners.
• This study shows that local anaesthetic systemic toxicity awareness is low, particularly among non-anaesthesiologists.
• Participants were supportive of the development of a mobile app to help them compute maximum safe doses.

## 1 Introduction

Local anaesthetics (LAs) are regularly used by anaesthesiologists and by many other specialists such as gynaecologists, surgeons, and emergency physicians [[1], [2], [3]].

The use of LAs is generally associated with few adverse effects [4]. However, if administered improperly and/or at a higher than recommended dose, local anaesthetic systemic toxicity (LAST) may occur. While uncommon, LAST can result in serious harm and even patient death [5,6]. Most often, however, LAST produces only mild, non-specific symptoms [7]. This certainly precludes the identification of many LAST cases and probably contributes to large differences in the incidence of LAST reported in the literature, ranging from 0.04 to 1.8/1000 [8].

Identification of LAST may also be hampered by a lack of knowledge about the existence, signs and symptoms of this complication [9,10]. There is however little available data regarding LAST knowledge and awareness among the different medical practitioners who use LAs on a regular basis.

The main objective of this study was to evaluate LAST knowledge and LA use among physicians from various medical specialties working in two Swiss university hospitals. The secondary objective was to determine whether these physicians felt the need for a specific tool to help them determine maximum safe LA doses.

## 2 Materials and methods

### 2.1 Study design

This was a closed web-based survey conducted in accordance with the Checklist for Reporting Results of Internet E-Surveys [11] between October 5th and November 2nd, 2022. A declaration of no objection was issued by the regional research ethics committee (Req-2021-00467) as this project did not fall within the scope of the Swiss Act on Research involving Human Beings [12].

### 2.2 Web-based platform

A Joomla! 3.10 platform (Open Source Matters Inc, New York, NY, USA) hosted on a Swiss server (https://survey.anesth.ch) was used to carry out this study. The Community Surveys component (version 5.9; Shondalai, BulaSikku Technologies Pvt, Hyderabad, Telangana, India) was used to create the web-based survey. Answers were automatically recorded in an encrypted MySQL-compatible database (MariaDB version 10.3; MariaDB Corporation Ab, MariaDB Foundation, Middletown, Delaware, USA). AcyMailing 7.9 (Acyba, Lyon, France) was used to manage email distribution lists.

The questionnaire consisted of 6 pages, some of which were not displayed depending on the participants' answers (Supplementary Table 1). This branching logic strategy also allowed for a shorter questionnaire when participants answered that they did not use LAs in their clinical practice. The maximum number of questions a participant was required to answer was 29, with a minimum of 11. These questions were validated by a panel of experts including a clinical pharmacologist and senior anaesthesiologists. Regular expression (RegEx) rules were used to avoid inconsistent answers. Randomization or alternation of survey items were not used as they were not deemed relevant given the study's objective and design. The questions about the risks of LA use were displayed on 3 different pages to prevent influencing participants' choices. The system ensured that participants had answered all relevant questions on a page before allowing them to move forward. All answers could be reviewed and changed before moving on to the next page but could not be changed afterwards.

Before launching the study, the web-based survey and data extraction mechanism were thoroughly tested by all investigators for usability, clarity, and technical functionality. The usefulness and comprehensiveness of the questions and answers provided were also assessed.

### 2.3 Participants and recruitment

The target population was composed of medical practitioners most likely to use LAs in two Swiss university hospitals (Hôpitaux Universitaires de Genève – HUG, Geneva; and Centre Hospitalier Universitaire Vaudois – CHUV, Lausanne). The medical specialties of interest were identified by the experts. Then, the appropriate medical Heads of Departments were contacted to obtain their agreement to access the email addresses of their employees. The secretaries of those who agreed (17/29, 58.6 %) were contacted to obtain these addresses, which were merged into a master list. This list was curated by removing double entries and email addresses belonging to the Medical Heads of Departments, to people other than medical professionals or to professionals who primarily worked in other hospitals. Email addresses nonspecific to the given university hospital were also removed. The remaining email addresses were added to an AcyMailing distribution list. Two of the Medical Heads of Departments who refused to give us access to individual email addresses nevertheless agreed to let their secretaries send a generic link to the study platform. Although such a link was indeed created, the lack of control over the distribution of emails and the clear reluctance of some secretaries to send regular reminders represented a significant deviation from the study protocol. Therefore, the records obtained through this generic link were not included in this analysis.

Emails inviting medical practitioners to participate in the study were sent on September 5th, 2022. These emails were identical except for the unique survey links which were automatically generated and contained unique, individual tokens. The use of these unique links prevented any double entry. The invitation email stated the purpose of the study and acknowledged its approval by the relevant Head of Department. The estimated time for completion, the name and contact of the principal investigator and a disclaimer containing a data policy statement were displayed both on the invitation email and on the welcome page of the survey (Fig. 1). Informed consent was gathered electronically. Participation was voluntary and no incentive was given to promote participation. Four reminders were sent during the study periods to encourage participation in the non-responding population (September 20th, October 3rd, October 17th and October 27th). The study website was put offline on November 2nd, 2022, thereby preventing any participation beyond this point.

**Fig. 1:**
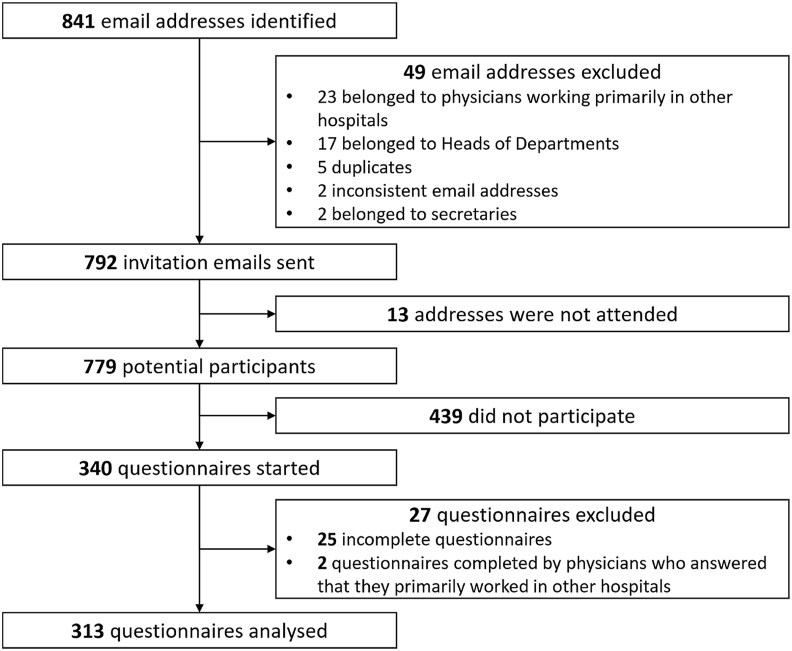
Study flowchart.

Given the design of this study, there was no predetermined sample size, and no sampling scheme was used. Therefore, the participants who completed the survey represent a convenience sample.

### 2.4 Outcomes

The primary outcome was the proportion of participants who identified “intoxication” (i.e., LAST) as a risk linked to LA use along with the two main systems involved (neurological and cardiovascular). Responses were considered incorrect when participants did not identify LAST as a risk, identified only one of the two systems involved, or answered that other systems (immune, digestive, urinary) were involved.

Secondary outcomes were the proportion of medical practitioners aware of LAST treatment, adapting LA doses according to weight, comorbidities, and current medications. The types of LAs used by respondents were also analysed along with LA use frequency. The tools used to compute LA doses were also assessed, as well as physicians’ perception of the calculation rules and their perceived need for a specific tool to compute safe LA doses.

### 2.5 Data curation and statistical analysis

Data were extracted from the database and imported into Stata (version 17.0; StataCorp LLC) for data curation. Records of participants who did not complete the first questionnaire were excluded. In accordance with FAIR (findability, accessibility, interoperability, and reuse) principles [13], the curated DTA file (Stata dataset file) is freely available on the Yareta repository [14].

Descriptive characteristics are reported using mean (SD) or median (Q1:Q3) depending on the variable of interest. When relevant, items derived from 6-point Likert scales were binarized. Given the sample size, only parametric tests were used (Chi-squared test for categorical variables, or Student t-test for continuous variables). Multivariable logistic regression was used to determine an association between knowledge of LAST and years of experience in the current medical specialty, age, country of diploma, frequency of LA use (daily vs less frequently), clinical position, and being an anaesthesiologist. These variables were determined a priori according to their clinical relevance.

## 3 Results

The participation rate was 40.2 % (313/779, Fig. 1). This rate was significantly higher among anaesthesiologists (154/249, 61.8 %) than among non-anaesthesiologists (159/530, 30.0 %; *P* < .001). Participant characteristics are detailed in Table 1. While some characteristics, such as age, are statistically significant, their actual relevance is probably limited.

**Table 1:** Characteristics of study participants.

Characteristics	Anaesthesiologists (N = 154)	Non-anaesthesiologists (N = 159)	*P* value
Gender (n, %)			
*Female*	68 (44.2 %)	74 (46.5 %)	.556
*Male*	85 (55.2 %)	85 (53.5 %)	
*Other*	1 (0.7 %)	0 (0 %)	
Age (years, mean, SD)	39 (±8)	36 (±8)	.002
Centre (n, %)			
*CHUV*	72 (46.8 %)	61 (38.4 %)	.133
*HUG*	82 (53.3 %)	98 (61.6 %)	
Country of diploma (n, %)			
*Switzerland*	102 (66.2 %)	110 (69.2 %)	
*Other*	52 (33.8 %)	49 (30.8 %)	.577
Years since diploma (mean, SD)	13 (±8)	10 (±7)	.002
Years of experience in current specialty (mean, SD)	9 (±8)	8 (±6)	.030
Position (n, %)			
*Resident*	62 (40.3 %)	69 (43.4 %)	
*Fellow*	54 (35.1 %)	53 (33.3 %)	
*Consultant*	38 (24.7 %)	37 (23.3 %)	.853
Medical divisions (n, %)			
*Anaesthesiology*	154 (100 %)		N/A^a^
*Emergency Department*		65 (40.9 %)	
*Gynaecology/Obstetrics*		35 (22.0 %)	
*Visceral surgery*		16 (10.1 %)	
*Orthopaedic surgery*		11 (6.9 %)	
*Plastic surgery*		7 (4.4 %)	
*Neurosurgery*		5 (3.1 %)	
*Other*		5 (3.1 %)	
*Hand surgery*		4 (2.5 %)	
*Thoracic surgery*		4 (2.5 %)	
*Maxillofacial surgery*		3 (1.9 %)	
*Cardio-vascular surgery*		2 (1.3 %)	
*Ophthalmology*		2 (1.3 %)	

Totals may not equal 100 % due to rounding.

a N/A: Not applicable.

**Fig. 2:**
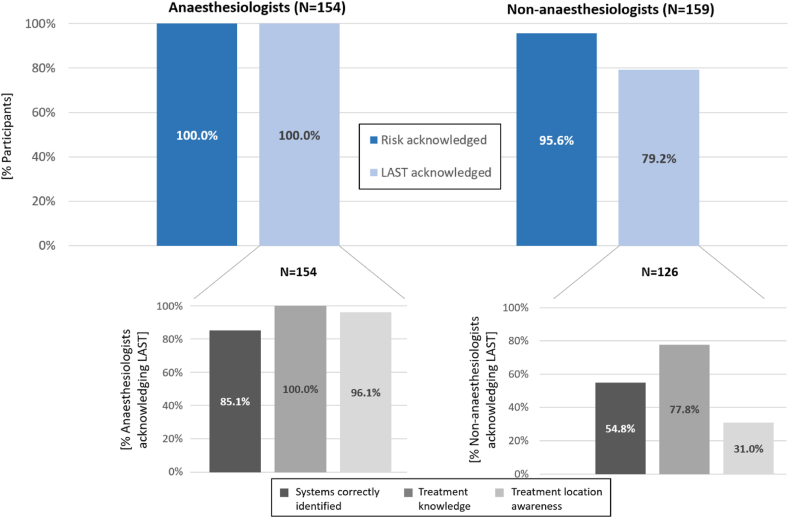
Knowledge of local anaesthetic systemic toxicity (LAST) and awareness of LAST treatment and treatment location.

Two participants answered that there were no risks associated with LA use (2/313, 0.6 %), while 5 answered that they did not know (1.6 %). Those who answered that there were no risks did not use LAs in their clinical practice. Nevertheless, four of those who answered that they did not know reported using such agents. These participants were all non-anaesthesiologists. While all anaesthesiologists acknowledged LAST as a risk (154/154, 100 %), this risk was only identified by 79.2 % of non-anaesthesiologists (126/159; *P* < .001; Fig. 2). Regarding the primary outcome, the proportion of participants who acknowledged LAST and correctly identified the systems involved was 63.9 % (200/313). This proportion was almost twice as high among anaesthesiologists (85.1 %, 131/154 vs 43.4 %, 69/159; *P* < .001). After adjusting for years of clinical experience, age, country of diploma, frequency of LA use, clinical position and being an anaesthesiologist, the only significant associations were this latter factor (*P* < .001) and clinical position, with fellows and consultants displaying higher knowledge levels (*P* = .016 and *P* = .046, respectively).

Among the participants who identified LAST as a risk, several non-anaesthesiologists declared not knowing how to treat this complication (28/126, 22.2 %) whereas no anaesthesiologists answered that they did not know (*P* < .001). In both groups, some participants answered they would not know where to find specific LAST treatment (6/154 anaesthesiologists, 3.9 % vs 87/126 non-anaesthesiologists, 69.1 %; *P* < .001). Fig. 2 further details these proportions.

**Fig. 3:**
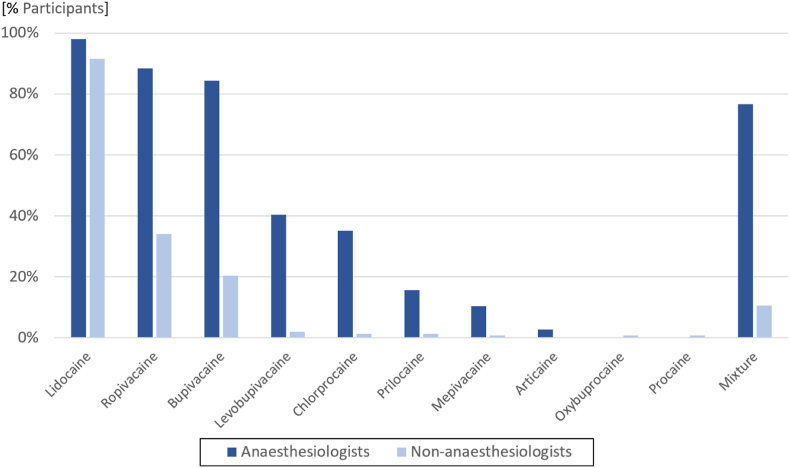
Use of specific local anaesthetics and mixtures among anaesthesiologists and non-anaesthesiologists.

Most participants answered that they used LAs in their clinical practice (307/313, 98.1 %). The majority reported using these agents on a daily basis (174/307, 56.7 %), with anaesthesiologists reporting higher utilization rates (*P* < .001). Anaesthesiologists declared using LA mixtures more frequently than non-anaesthesiologists (118/154, 76.6 % vs 16/153, 10.5 %; *P* < .001). The 3 most frequently used LAs were lidocaine (291/307, 94.8 %), ropivacaine (188/307, 61.2 %) and bupivacaine (161/307, 52.4 %) (Fig. 3). The median number of LAs used by non-anaesthesiologists was of 1 (1:2), whereas the median was of 4 (3:5) among anaesthesiologists (*P* < .001).

Most anaesthesiologists responded that they adapted LA doses often, very often or always (142/154, 92.2 %), whereas only a minority of non-anaesthesiologists did (41/153, 26.8 %; *P* < .001; Fig. S1 - Frequency of local anaesthetic dose adaptation between anaesthesiologists and non-anaesthesiologists).

Among those who answered that they adapted LA doses (even rarely), 88.2 % (135/153) of anaesthesiologists reported that they performed mental calculations often, very often or always, versus 48.4 % (45/93) of non-anaesthesiologists (*P* < .001). Participants reported an infrequent use of calculators, with only 34.6 % (53/153) of anaesthesiologists answering that they used them often, very often or always, versus 8.6 % (8/93) of non-anaesthesiologists (*P* < .001). Many non-anaesthesiologists (39.8 %, 37/93) answered that they asked a colleague to help them adapt the dose, while only few anaesthesiologists resorted to such help (5/153, 3.3 %; *P* < .001).

**Fig. 4:**
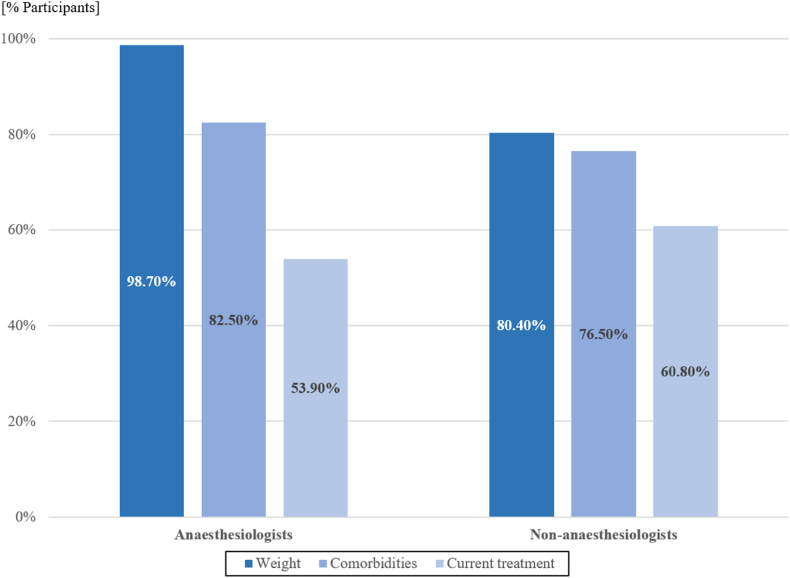
Proportion of anaesthesiologists and non-anaesthesiologists who responded that they adapted local anaesthetic doses at least often according to specific patient characteristics.

Weight was considered as the main factor to take into consideration to adapt LA doses (275/307, 89.6 %), followed by patient comorbidities (244/307, 79.5 %) and current treatments (176/307, 57.3 %). The proportion of practitioners who answered that they adapted LA doses at least often according to these characteristics is detailed in Fig. 4.

Most anaesthesiologists agreed at least somewhat that LA calculation rules were clear (117/154, 76.0 %). However, only 43.8 % (67/153) of non-anaesthesiologists shared this opinion (*P* < .001) (Fig. S2 - Perception of whether practitioners felt that local anaesthetic dose calculation rules were clear).

Most respondents agreed that a tool designed to help them compute maximum safe LA doses would be useful (251/313, 80.2 %). Most were in favour of a mobile app (190/251, 75.7 %). More than half were in favour of a tool integrated in the electronic patient record (129/251, 51.4 %), while only few were supportive of a software accessible on their hospital's network (40/251, 15.9 %). Analog tools were not endorsed by the respondents: only 35.1 % (88/251) were in favour of guidelines posted in the workplace and even less (37/251, 14.7 %) were supportive of flash cards.

## 4 Discussion

This web-based study shows that LAST knowledge deserves to be improved among medical practitioners. Indeed, more than a third of all respondents answered incorrectly according to the definition of our primary outcome. While this proportion was significantly lower among anaesthesiologists, some still answered incorrectly and many responded that they did not adapt LA doses according to current patient treatment and even to other important factors, thereby potentially administering unsafe LA doses. This is a cause for concern as over 20 % of non-anaesthesiologists reported using bupivacaine, which is associated with a particularly high risk of LAST [15,16].

Calculating safe LA doses is no simple feat. Prior studies have shown that calculation rules are mostly inconsistent and often result in difficult and time-consuming computations [17,18]. Nevertheless, in the present study, most anaesthesiologists thought that these rules were rather clear. Whether clear rules are indeed used by these specialists to compute maximum safe LA doses remains to be established, but the lack of LA dose adaptation according to current treatments or even to other relevant factors such as weight and comorbidities is concerning.

Many non-anaesthesiologists seem to rely on their colleagues to assess maximum safe LA doses. Since these colleagues are most likely anaesthesiologists, it is all the more important for these professionals to have a reliable, fast and easily accessible tool to compute these doses. However, despite the many factors which should be taken into account to compute safe LA doses [19,20], the majority of anaesthesiologists responded that they resorted to mental calculation, with only a minority reporting the use of calculators. The fact that most respondents were in favour of the development of a tool designed to help them compute maximum safe LA doses, and particularly of a mobile app, shows that a need for a reliable and user-friendly tool indeed exists. Among such tools, a recently developed mobile app, called LoAD Calc for Local Anaesthetic Dose Calculator [21], could prove particularly useful and deserves to be further studied.

Other solutions may be considered to help healthcare professionals calculate maximal safe LA doses. A potentially efficient solution could be to integrate calculators directly in the patient's electronic record, thereby allowing these calculators to take all known (and relevant) parameters into account [22]. The ever increasing deployment of artificial intelligence (AI) in anaesthesiology could also lead to the development of AI-based solutions [23]. Such tools must however be carefully assessed prior to their deployment in the OR since AI, and particularly generative AI, can be prone to hallucinations [24].

This study has some limitations. First, even though the participation rate was rather high, participants were only recruited among two Swiss university hospitals. Therefore, these results might not apply to regional hospitals, or even to other countries. This latter issue is however mitigated by the fact that more than 30 % of participants graduated abroad, and there was no association between graduating from a country other than Switzerland and LAST knowledge according to multivariable logistic regression. In the same line, this survey focused on medical specialties considered particularly likely to use LAs. A wider assessment of LA use, including other medical specialties, could therefore be interesting to carry out. In addition, LA knowledge among non-physicians was not assessed in the course of this study. It might however be interesting to obtain such data, since other healthcare professions sometimes administer LAs autonomously (e.g., midwives who often manage epidural analgesia). Finally, the sample size was quite small. This could be due to the fact that some Medical Heads of Departments refused to provide the email addresses of their employees. However, the present study was able to assess more than 12 different specialties other than anaesthesiology with a proper response rate.

Some strengths should be acknowledged. The high participation rate supports the validity of these results. In addition, the fully self-standing survey platform and its independence from third-parties such as Google, Microsoft, or other survey vendors ensured the reliability and independence of the collected data. In addition, the use of unique tokens prevented duplicate entries and may have contributed to limit attrition by allowing participants to resume the survey at will [25].

Apart from the need for a specific tool designed to help medical practitioners compute maximum safe LA doses, these results highlight the importance of educational needs in terms of knowledge and appreciation of LA use risks. Future studies should therefore focus on these two elements.

## 5 Conclusions

LAST awareness is limited among practitioners who use LAs on a regular basis. Educational interventions regarding this risk should be created and tools designed to help calculate maximum safe LA doses developed. In addition, future studies should assess the frequency of unsafe LA doses administration.

## Ethics statement

A declaration of no objection (2021–00467) was issued by the Commission cantonale d’éthique de la recherche (CCER, Geneva, Switzerland).

## Human and animal rights

The authors declare that the work described has been carried out in accordance with the Declaration of Helsinki of the World Medical Association revised in 2013 for experiments involving humans as well as in accordance with the EU Directive 2010/63/EU for animal experiments.

Not applicable.

## Informed consent and patient details

The authors declare that this report does not contain any personal information that could lead to the identification of the patient(s).

Not applicable.

The authors declare that they obtained a written informed consent from the patients and/or volunteers included in the article. The authors also confirm that the personal details of the patients and/or volunteers have been removed.

## Disclosure of interest

The authors declare that they have no known competing financial or personal relationships that could be viewed as influencing the work reported in this paper.

The authors declare the following financial or personal relationships that could be viewed as influencing the work reported in this paper.

## Funding

This work did not receive any grant from funding agencies in the public, commercial, or not-for-profit sectors.

## CRediT authorship contribution statement

**Tal Sarah Beckmann:** Writing – review & editing, Writing – original draft, Investigation, Formal analysis. **Caroline Flora Samer:** Writing – review & editing, Conceptualization. **Hannah Wozniak:** Writing – review & editing, Formal analysis, Data curation. **Georges Louis Savoldelli:** Writing – review & editing, Methodology, Conceptualization. **Mélanie Suppan:** Writing – review & editing, Writing – original draft, Validation, Supervision, Software, Resources, Project administration, Methodology, Investigation, Formal analysis, Data curation, Conceptualization.

## Declaration of competing interest

The authors declare that they have no known competing financial interests or personal relationships that could have appeared to influence the work reported in this paper.

## Figures and Tables

**figs1:**
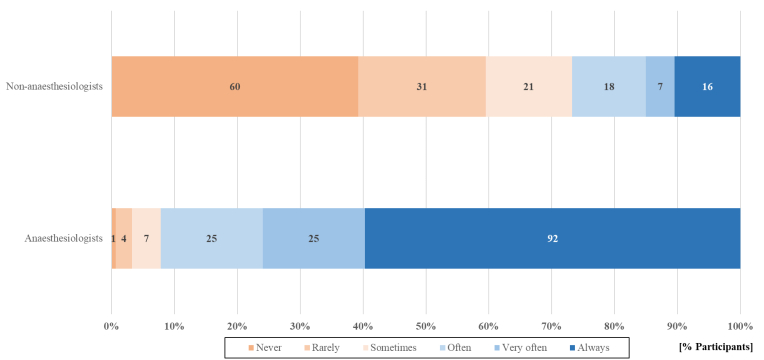


**figs2:**