# Supplementary material for: Local anaesthetics risks perception: A web-based survey
Source: Heliyon. 2023 Dec 13;10(1):e23545. doi: 10.1016/j.heliyon.2023.e23545 (PMC10770561; doi:10.1016/j.heliyon.2023.e23545)
Supplement: Multimedia component 1 [file mmc1.docx]

**Supplementary Table 1. Questionnaire.**

## Page 1 – Demographics

| **Page** | **#** | **Original Question (FR)** | **Translated Version (EN)** | **Comments** |
| --- | --- | --- | --- | --- |
| 1 | 1 | Dans quelle institution travaillez-vous principalement?   - Hôpitaux Universitaires de Genève (HUG) - Centre Hospitalier Universitaire Vaudois (CHUV) - Autre | In which institution do you primarily work?   - Geneva University Hospitals (HUG) - Lausanne University Hospital (CHUV) - Other | A free text field was displayed to those who answered “Other”. |
|  | 2 | Dans quel service travaillez-vous principalement?   - Anesthésiologie - Chirurgie cardiaque/vasculaire - Chirurgie de la main - Chirurgie maxillo-faciale - Chirurgie plastique - Chirurgie thoracique - Chirurgie viscérale - Dermatologie - Gynécologie/obstétrique - Médecine dentaire - Neurochirurgie - Ophtalmologie - ORL - Orthopédie - Urgences - Autre | In which department do you primarily work?   - Anaesthesiology - Cardiac/Vascular surgery - Hand surgery - Maxillofacial surgery - Plastic surgery - Thoracic surgery - Visceral surgery - Dermatology - Gynaecology/Obstetrics - Dental medicine - Neurosurgery - Ophthalmology - ENT - Orthopaedic surgery - Emergency - Other | A free text field was displayed to those who answered “Other”. |
|  | 3 | Quelle est votre fonction?   - Médecin assistant(e) / interne - Chef(fe) de clinique - Médecin adjoint(e) / médecin cadre - Autre | What is your position?   - Resident - Fellow - Consultant - Other | A free text field was displayed to those who answered “Other”. |
|  | 4 | En quelle année avez-vous obtenu votre diplôme de médecin? | In what year did you graduate as a physician? | A regular expression (RegEx) rule was used to ensure that only years between 1950 and 2022 could be entered. |
|  | 5 | Dans quel pays avez-vous obtenu votre diplôme de médecin?   - Suisse - Autre | In which country did you obtain your medical degree?   - Switzerland - Other | A free text field was displayed to those who answered “Other”. |
|  | 6 | Vous avez répondu Suisse à la question précédente, pouvez-vous préciser dans quel canton vous avez obtenu votre diplôme?   - Bâle - Berne - Genève - Lausanne - Zürich | You answered Switzerland in the previous question, can you specify in which canton you obtained your degree?   - Basel - Bern - Geneva - Lausanne - Zürich |  |
|  | 7 | Combien d'années d'expérience avez-vous dans votre spécialité? | How many years of experience do you have in your specialty? | A regular expression (RegEx) rule was used to ensure that only numbers between 0 and 50 could be entered. |
|  | 8 | Quel est votre genre?   - Homme - Femme - Autre | What is your gender?   - Man - Woman - Other |  |
|  | 9 | Quel est votre âge? | How old are you? | A regular expression (RegEx) rule was used to ensure that only numbers between 0 and 50 could be entered. |

## Page 2– Local Anaesthetics Use

| **Page** | **#** | **Original Question (FR)** | **Translated Version (EN)** | **Comments** |
| --- | --- | --- | --- | --- |
| 2 | 10 | Utilisez-vous des anesthésiques locaux dans le cadre de votre spécialité?   - Oui - Non | Do you use local anaesthetics in your specialty?   - Yes - No | Participants who answered “No” to this question were redirected to page # |
|  | 11 | A quelle fréquence utilisez-vous des anesthésiques locaux?   - Moins qu'une fois par mois - Une à deux fois par mois - Une à deux fois par semaine - Une à deux fois par jour - Plus que deux fois par jour | How often do you use local anaesthetics?   - Less than once a month - Once or twice a month - Once or twice a week - Once or twice a day - More than twice a day | This question was only displayed to participants who answered “Yes” to question 10. |
|  | 12 | Parmi les anesthésiques locaux suivants lesquels utilisez-vous fréquemment dans votre pratique?   - Articaïne - Bupivacaïne - Chlorprocaïne - Lévobupivacaïne - Lidocaïne - Lidocaïne + adrénaline - Mepivacaïne - Prilocaïne - Ropivacaïne - Autre | Which of the following local anaesthetics do you use frequently in your practice?   - Articaine - Bupivacaine - Chlorprocaine - Levobupivacaine - Lidocaine - Lidocaine + Adrenaline - Mepivacaine - Prilocaine - Ropivacaine - Other | This question was only displayed to participants who answered “Yes” to question 10.  A free text field was displayed to those who answered “Other”. |
|  | 13 | Vous arrive-t-il d’utiliser un mélange de différents anesthésiques locaux?   - Oui - Non | Do you ever use mixtures of different local anaesthetics?   - Yes - No |  |
|  | 14 | Vous arrive-t-il d'adapter la dose d'anesthésique local en fonction des caractéristiques de vos patient(e)s?   - Jamais - Rarement - Parfois - Souvent - Très souvent - Toujours | Do you ever adapt the dose of local anaesthetic to patient characteristics?   - Never - Rarely - Sometimes - Often - Very often - Always |  |
|  | 15 | Comment effectuez-vous cette adaptation de la dose d'anesthésique local?   - Je calcule de tête la dose à administrer pour chaque patient(e) - Je calcule avec une calculatrice la dose à administrer pour chaque patient(e) - Je calcule grâce à un autre outil la dose à administrer pour chaque patient(e) - Mes collègues m'indiquent la dose à administrer | How do you adjust the dose of local anaesthetic?   - I perform a mental calculation of the dose to be administered for each patient - I use a calculator to determine the dose for each patient - I use another tool to calculate the dose for each patient - My colleagues tell me the dose to administer | This question was only displayed to participants who did not answer “Never” to question 14.  For each item, a 6-point scale identical to that used in question 14 (“Never” 🡺 “Always”) was displayed.  A free text field was displayed when participants answered that they used another tool. |
|  | 16 | Lors de l'administration d'anesthésiques locaux chez des patient(e)s adultes:   - Il faut effectuer un test d'aspiration avant d'injecter l'anesthésique local - Il faut adapter la dose au poids du (de la) patient(e) - Il faut adapter la dose si le (la) patient(e) a des comorbidités importantes - Il faut adapter la dose si le (la) patient(e) prend régulièrement certains médicaments - Les règles pour le calcul de la dose administrable sont claires | When administering local anaesthetics to adult patients:   - An aspiration test should be performed before injecting the local anaesthetic - The dose should be adjusted to the patient's weight - The dose should be adjusted if the patient has significant comorbidities - The dose should be adapted if the patient is regularly taking certain medications - The rules for calculating the dose to be administered are clear | For each item, a 6-point scale was used:   1. Strongly disagree 2. Disagree 3. Somewhat disagree 4. Somewhat agree 5. Agree 6. Strongly agree |

## Pages 3-5 – Local Anaesthetics Risks

| **Page** | **#** | **Original Question (FR)** | **Translated Version (EN)** | **Comments** |
| --- | --- | --- | --- | --- |
| 3 | 17 | Pensez-vous qu'il existe des risques liés à l'utilisation des anesthésiques locaux?   - Oui - Non - Je ne sais pas | Do you think there are any risks associated with the use of local anaesthetics?   - Yes - No - I do not know | Those who answered “No” proceeded directly to page 6. |
| 4 | 18 | Quels sont les risques liés à l'utilisation des anesthésiques locaux? (Plusieurs réponses possibles)   - Hyperthermie maligne - Infection - Intoxication - Lésion nerveuse - Nausées et vomissements - Réaction allergique | What are the risks associated with the use of local anaesthetics? (More than one possible answer)   - Malignant hyperthermia - Infection - Intoxication - Nerve injury - Nausea and vomiting - Allergic reaction |  |
| 5 | 19 | Quels sont les systèmes atteints lors d'une intoxication aux anesthésiques locaux? (plusieurs réponses possibles)   - Le système digestif - Le système nerveux - Le système immunitaire - Le système cardio-vasculaire - Le système urinaire | Which systems are affected by local anaesthetic toxicity? (more than one possible answer)   - The digestive system - The nervous system - The immune system - The cardiovascular system - The urinary system | This question was asked regardless of the answers given on page 4. |
|  | 20 | Le traitement de l'intoxication aux anesthésiques locaux est:   - Un traitement de support - Un traitement spécifique - Les deux (traitement spécifique et traitement de support) - Je ne sais pas | Treatment of local anaesthetic toxicity is:   - Supportive - Specific - Both (specific and supportive) - I do not know |  |
|  | 21 | Savez-vous où se trouve dans votre hôpital le matériel nécessaire pour traiter une intoxication aux anesthésiques locaux?   - Oui - Non | Do you know where to find the necessary supplies to treat local anaesthetic toxicity in your hospital?   - Yes - No |  |

## Page 6 – Tools

| **Page** | **#** | **Original Question (FR)** | **Translated Version (EN)** | **Comments** |
| --- | --- | --- | --- | --- |
| 6 | 22 | Pensez-vous qu'un outil permettant de calculer la dose administrable d'anesthésiques locaux serait utile?   - Oui - Non | Do you think a tool to calculate the safe dose of local anaesthetics would be useful?   - Yes - No |  |
|  | 23 | Vous avez répondu oui à la question précédente. Pouvez-vous préciser quel type d'outil serait le plus utile?   - Incorporé dans le dossier patient informatisé (ou dans l'outil informatique que vous utilisez habituellement) - Nouvel outil informatique accessible sur le réseau hospitalier - Application mobile (sur smartphone) - Outil papier personnel (pense-bête, aide-mémoire, carnet) - Fiche d'attitude affichée dans le lieu de travail - Autre | You agreed to the previous question. Can you specify which type of tool would be most useful?   - Incorporated into the computerized patient record (or into the computerized tool you usually use) - New IT tool accessible on the hospital network - Mobile application (on smartphone) - Personal paper tool (reminder, memory aid, notebook) - Action card posted in the workplace - Other | This question was only displayed to participants who answered “Yes” to question 22.  A free text field was displayed to those who answered “Other”. |
